# Identification of a C_2_H_2_ Transcription Factor (PsCZF3) Associated with RxLR Effectors and Carbohydrate-Active Enzymes in *Phytophthora sojae* Based on WGCNA

**DOI:** 10.3390/jof8100998

**Published:** 2022-09-22

**Authors:** Yanhong Hu, Zhihua He, Yebin Kang, Wenwu Ye, Linkai Cui

**Affiliations:** 1 College of Horticulture and Plant Protection, Henan University of Science and Technology, Luoyang 471000, China; 2 Department of Plant Pathology, Nanjing Agricultural University, Nanjing 210095, China

**Keywords:** digital RNA-seq, *Phytophthora sojae*, WGCNA, transcription factor, RxLR effector

## Abstract

*Phytophthora sojae* is a destructive soybean pathogen that orchestrates various secreted proteins (effectors) to modulate plant immunity and facilitate infection. Although a number of effectors have been identified and functionally studied in *P. sojae*, the way these molecules are regulated is marginally known. In this study, we performed a weighted gene correlation network analysis (WGCNA) based on digital RNA-seq, which enabled the identification of a transcription factor (PsCZF3) in *P. sojae*. This transcription factor is a C_2_H_2_-type zinc finger protein that regulates the transcription of 35 RxLR effectors during the early infection stage. Phylogenetic analysis revealed that PsCZF3 is a highly conserved protein across oomycetes, suggesting that this regulation mechanism may broadly exist in oomycete species. In addition, by building a subnetwork of *PsCZF3* and correlated genes, we also found that PsCZF3 contributed to the transcriptional regulation of carbohydrate-active enzymes. Our findings suggest that the activation of *PsCZF3* facilitates *P. sojae* infection by up-regulating RxLR effectors and carbohydrate-active enzymes.

## 1. Introduction

*Phytophthora sojae* is a devastating oomycete pathogen that causes the stem and root rot of soybean crop, resulting in approximately USD1–2 billion losses globally per year [1]. Generally, oomycete pathogens use different mechanisms, such as the production of extracellular toxins, hydrolytic enzymes and inhibitors, and effector proteins, for their virulence [2]. Particularly, the proteaceous effectors, which are one of the most important virulence factors, target numerous host cellular processes, commonly leading to the suppression of PAMP-triggered immunity (PTI), effector-triggered immunity (ETI), and programmed cell death (PCD) [2]. Thus far, the best-studied effector class in oomycetes is the RxLR effectors, which are named for a conserved N-terminal amino acid sequence motif (arginine, any amino acid, leucine, arginine) [2]. Genomic and transcriptomic studies have advanced our understanding of the biology and pathology of *P. sojae* RxLR effectors. With genomic analysis, the *P. sojae* reference genome was found to have approximately 400 genes encoding RxLR effectors [3,4]. Transcriptomic analyses further indicated that these effectors were expressed in a timely manner. The immediate-early effectors were strongly expressed before infection and moderately induced upon infection (2- to 10-fold), while the early effectors were very weakly expressed prior to infection, but strongly induced (10- to 120-fold) during the first 12 h of infection patterns [4,5]. To date, however, the way these effectors are regulated is unknown.

In eukaryotes, the regulation of gene expression generally depends on the activity of trans acting factors such as transcription factors, which subsequently regulate the transcriptional initiation of genes. Transcription factors can be involved in the transcriptional regulation of different (multiple) genes [6]. In oomycetes, transcription factors have been reported to contribute to pathogen development and virulence. For instance, in both *P. sojae* and *P. infestans*, the MADS-box transcription factors played significant roles in sporulation [7,8], while the Myb transcription factors regulated both vegetative growth and sporulation [9,10]. Other transcription factors have been shown to be involved in the resistance of plant defense response [11,12,13]. Despite the association of transcription factors with pathogenesis that have been found, no transcription factor has been reported to directly regulate RxLR effectors.

Weighted gene co-expression network analysis (WGCNA) is a powerful network analysis tool that describes the correlation of gene expression based on the microarray or RNA-seq data. It takes advantage of a graph theoretical approach to understanding correlations amongst genes and grouping genes into modules that typically have coordinated biological functions and regulatory mechanisms [14]. Currently, WGCNA has been extensively used to construct gene co-expression networks and identify centrally connected hub genes in humans, animals and plants [14,15,16]. However, this method is rarely applied to plant pathogens.

The objective of this study was to identify transcription factors associated with RxLR effectors by WGCNA. In the present study, the WGCNA was constructed based on the digital RNA-seq data during the early stage of the *P. sojae* infection. Key gene modules including a large number of RxLR effectors were identified. In one key module, we found a transcription factor closely associated with RxLR effectors and carbohydrate-active enzymes. The structure, DNA-binding sites and conservation of the protein were analyzed in detail. Based on the bioinformatic analyses, our study is expected to provide novel insights into the transcriptional regulation mechanisms of RxLR effectors.

## 2. Materials and Methods

### 2.1. Strain and Culture Conditions

*P. sojae* strain P6497 was used in this study. The strain was grown in 20% V8 liquid medium at 25 °C under darkness for 72 h and was then washed with sterile distilled water in order to collect the culture. The soybean cultivar Williams, which is susceptible to P6497, was grown in a greenhouse at 25 to 30 °C and was used at the second-leaf stage. A mycelial mat (d = 3 cm) was laid on and sandwiched between the upper surfaces of two leaves at 25 °C, respectively, for 0, 6, 12, 24 and 48 h post-inoculation (hpi) [5]. Three biological replicates were applied for each time point. The regions of the leaves in contact with the mycelia were excised together with the mycelia and were preserved in liquid nitrogen.

### 2.2. RNA Isolation, Library Preparation, and Sequencing

Total RNA was isolated from a mixture of leaf and mycelium using TRIzol reagent (Thermo Scientific, Waltham, MA, USA) according to the manufacturer’s recommendation. The quality of RNA was estimated by using 1% agarose gel electrophoresis, and its concentration and purity were assessed by using the NanoDrop 2000 Spectrophotometer (Thermo Scientific, Waltham, MA, USA). The integrity of total RNA were assessed on the Agilent Bioanalyzer 2100 system (Agilent Technologies, Palo Alto, CA, USA) using the RNA Nano 6000 Assay Kit. The sequencing library was constructed with KC-Digital Stranded mRNA-seq Library Prep Kit for Illumina (Seqhealth, Wuhan, China), according to the manufacturer’s instructions. Oligo (DT) magnetic beads were used to enrich the mRNA with poly-A structure in the total RNA. The RNA was fragmented into ~300-bp fragments by ion interruption. Purified RNA was reversely transcribed into cDNA using random primers and reverse transcriptase, and the unique identifier (UID; a short sequence of 8–10 nt) was added. After the construction of the library, PCR was conducted to enrich the library fragments. Finally, the fifteen libraries were sequenced using the Illumina Hiseq X Ten System.

### 2.3. Digital RNA-Seq and Data Analysis

Using the Illumina Hiseq X Ten System, transcriptome sequencing generated 150-bp paired-end (PE) raw reads. The raw reads were subjected to quality control. High-quality reads were filtered using Trimmomatic v0.36 (Anthony M. Bolger, Berlin, Germany) [17], and data quality scores were sorted using the same software. The cleaned reads were further processed with the kcUID to remove duplication bias introduced during amplification and sequencing [18]. The consequent reads were grouped based on their UIDs, in which reads carrying the same UID were clustered into the same cluster. In the same cluster, the reads with a minimum of 95% sequence identity were extracted to generate a new sub-cluster through pairwise alignment. After all sub-clusters were generated, multiple sequence alignment was conducted to obtain an consensus sequence for every sub-cluster. The kcUID reads were mapped onto the *P. sojae* genome V3.0 using Hisat2 v2.0.1 (Daehwan Kim, Baltimore, MD, USA) [19]. The read count values on each gene as the original expression of the gene were compared using RSEM v1.2.31 (http://deweylab.github.io/RSEM/, accessed on 4 June 2016), and the expressions were standardized by RPKM (reads per kilobase per million mapped reads).

The gene expression profiles of all 15 samples were compared using the hierarchical clustering analysis (HCA). HCA was implemented according to a distance matrix of the Spearman correlation [20], and samples with a similar expression profile were clustered together. HCA was performed using the gplots functions in the R package [21]. The genes with a difference of at least two-fold change with *p*-value < 0.05 were considered as significantly differentially expressed genes. The “DESeq2” R language package was used to identify differently expressed genes (DEGs) between non-infection (mycelia) stages and infection stages [22].

### 2.4. Weighted Gene Co-Expression Network Analysis

Co-expression analysis was conducted using WGCNA [23]. A matrix of pairwise Pearson correlation coefficients between all pairs of genes was created, and the matrix was converted into an adjacency matrix using the soft threshold power beta (β), which proposes covariant similarity, and the topological overlap measure (TOM) similarity algorithm was used to transform the adjacency matrix into a topological overlap matrix to reduce noise and false correlation. Successively, hierarchical clustering was performed to identify modules. To obtain moderately sized modules, the minimum number of genes was set at 30 and merging modules with highly correlated (r > 0.75) eigengenes (defined as the first principal component of a given module and may be considered as a representative of the gene expression profiles in that module). All modules depicted in the hierarchical clustering dendrogram are henceforth referred to by their color labels. The grey module is used to hold all genes that do not clearly belong to any other module. To identify the relationship between modules and various infection stages, module–trait associations were estimated using the correlation between the module eigengene and the phenotype at the infection stages, which enabled the easy identification of the expression set (module) highly correlated with the phenotype. In addition, from the above analysis, we gained the weighted values, which represent the relationships between genes in pairs. The weighted values, which are >0.15, were used to perform network analysis using Cytoscape v3.7.2 (Paul Shannon, Seattle, WA, USA) [24]. Weighted values > 0.15 were considered highly correlated and strongly regulatory.

### 2.5. Gene Ontology (GO) Enrichment Analysis

The ClusterProfiler package of R was used to identify the GO categories significantly enriched with the differentially expressed genes that were highly correlated with PsCZF3 [25]. *p* < 0.05 was considered as a threshold of GO enrichment analysis.

### 2.6. DNA-Binding Site Prediction

In this study, two transcription factors (PsCZF3 and PsCZF4) were identified. Because PsCZF3 had stronger associations with RxLR effectors than PsCZF4, we focused on PsCZF3. The 0DNA-binding site prediction for the Cys2-His2 zinc finger transcription factor PsCZF3 was performed based on the webtools “ZFModels” (http://stormo.wustl.edu/ZFModels/, accessed on 1 April 2014) and “DNA-binding Specificities of Cys2His2 Zinc Finger Proteins” (http://zf.princeton.edu, accessed on 9 September 2010). The predicted DNA-binding sequence was represented as a sequence logo [26].

### 2.7. Identification and Phylogenetic Analysis of PsCZF3 Orthologs

PsCZF3 orthologs were selected based on Blastp searches against NCBI (threshold values were set to ≥80% coverage, ≥50% identity, and ≥200Max score). The full-length protein sequences of PsCZF3 orthologs were selected for phylogenetic analysis, and the tree was constructed by MEGA 6.0 [27], employing the neighbor-joining (NJ) algorithm. Bootstrap analysis with 1000 replicates was used to evaluate the significance of the nodes. Conserved motifs in the PsCZF3 homologous proteins were analyzed by MEME (https://meme-suite.org/meme/tools/meme, accessed on 5 February 2021) [28].

### 2.8. Validation by Reverse Transcription-Quantitative PCR (RT-qPCR)

The remaining total RNA samples were reversely transcribed to cDNA after sequencing libraries were constructed. Twelve genes from different modules were randomly selected for RT-qPCR analysis. Gene-specific primer pairs of the selected genes (Table S1) were designed using the primer design website (https://www.ncbi.nlm.nih.gov/tools/primer-blast/, accessed on 18 June 2012) and were commercially synthesized by Sangon Biotech Co., Ltd. (Shanghai, China). RT-qPCR was performed using a CFX96 Real-Time PCR Detection System (Bio-Rad, Hercules, CA, USA). The reaction mixture with a total volume of 20 µL contained 10 µL of 2XWiz Universal SYBR qPCR Master Mix, 2 µL of cDNA mix, 0.4 µL of each primer (10 µM), and 7.2 µL of RNase-free double-distilled H_2_O. The qRT-PCR was programmed at 95 °C for 60 s, followed by 40 cycles of 95 °C for 10 s and 60 °C for 20 s. A melting curve analysis was performed at the end of each PCR reaction at 95 °C for 15 s, 60 °C for 60 s, and 95 °C for 15 s. The *P. sojae* actin gene (ACT, accession number: XM_009530461.1) was used as a reference. Relative expression level was measured by normalizing the expression of targets against ACT, based on the 2^−ΔΔCT^ method [29]. RT-qPCR assay was repeated three times and three technical replicates were used for each RT-qPCR.

## 3. Results

**Figure 1:**
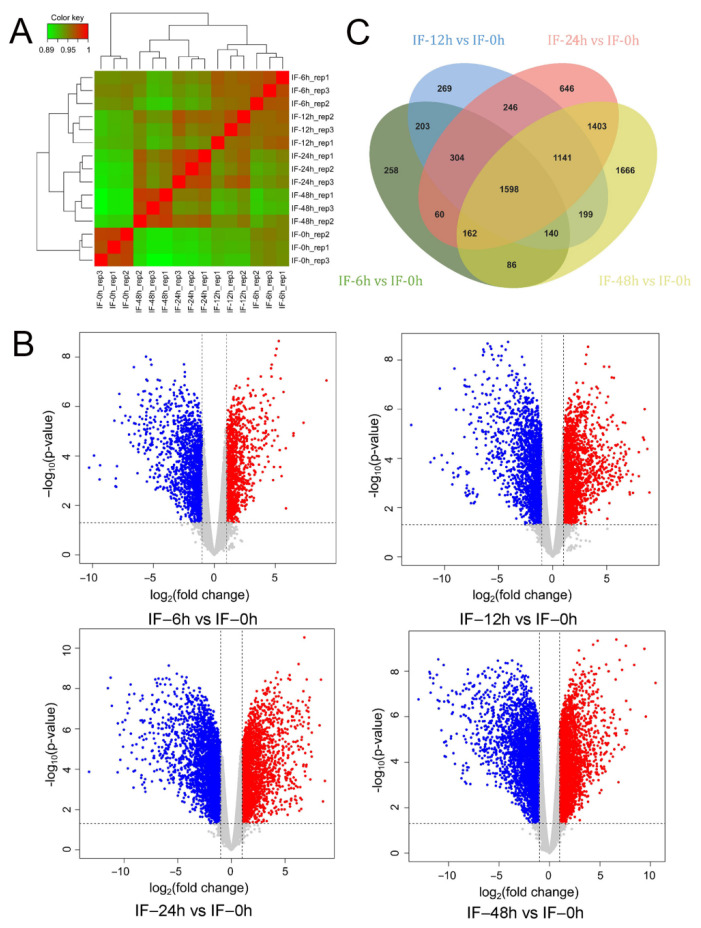
Comparison of gene expression and differentially expressed genes (DEGs) during the early infection stage of *P. sojae* to soybean leaves. (**A**) Hierarchical clustering analysis (HCA) of transcriptome data from 15 samples during the early infection. Color scale ranging from green to red indicates the inter-sample correlation from low to high. (**B**) Volcano plots illustrating the DEGs at different sampling time points after the inoculation of *P. sojae* to leaves. (**C**) Venn diagram showing the DEGs identified from comparison of 6, 12, 24 and 48 hpi with 0 hpi.

To create a weighted gene co-expression network and explore the transcription factors involved in the regulation of RxLR effectors, we performed the digital RNA-seq of the reference *P. sojae* strain P6497 during the early infection stage. The mycelium was sampled for RNA extraction at 0, 6, 12, 24, and 48 h after inoculation onto susceptible soybean leaf tissues. An average of 82,385,841 raw reads for a sample were generated (Table S2). To analyze the digital RNA-seq data, the raw sequencing reads were first filtered, which yielded 1112 million clean reads (Table S2). For each of the fifteen libraries, at least 98.40% of the clean reads had a quality score of Q30. The cleaned reads were further processed with the kcUID. Eventually, an average of 59 million of the UID reads were selected, and 89.79–97.00% of the reads were uniquely mapped to the *P. sojae* reference genome V3.0 (Table S3). In total, there were 28,142 genes, including both protein and non-protein coding genes. After excluding 4335 genes that showed an RPKM value = 0 in all 15 samples, 23,807 (84.6%) genes were present in at least one library. To test the correlation of the gene expression level among the samples, we conducted a hierarchical clustering analysis (HCA). Fifteen samples were clearly clustered into five groups on the basis of the infection time points, suggesting that there was a high correlation coefficient within the groups (Figure 1A).

To examine the DEGs, we compared the gene expression levels at 6, 12, 24, and 48 hpi to that at 0 hpi. Some 8381 DEGs were observed from the four different expression groups (Figure 1B,C). To validate the DEGs that were identified, we randomly picked 12 genes based on their expression patterns at the five infection time points, and conducted RT-qPCR analysis. The RT-qPCR results show a consistency with those of RNA-seq (Supplementary Figure S1), confirming the gene expression differentiation that we obtained from the DESeq2 analysis.

To determine the average expression pattern of RxLR effectors at each time point of early infection, we measured the expression level values (RPKM) for each infection group by pooling all RxLR effectors. A total of 316 RxLR effector genes were detected by the digital RNA-seq. The expression of these RxLR effectors was markedly up-regulated and reached the peak value at 12 hpi, and then gradually decreased over infection time (Supplementary Figure S2). Among the detected RxLR effector genes, 93 genes were differentially expressed. Further analysis of the differentially expressed RxLR effectors revealed that the expression of these genes changed more significantly at each time point during the early infection (Supplementary Figure S2), compared to the average expression of all RxLR effectors. Remarkably, the average expression level of the RxLR effectors at 12 hpi was significantly higher than those at other time points, implying that these genes may play a very important role in 12 hpi.

**Figure 2:**
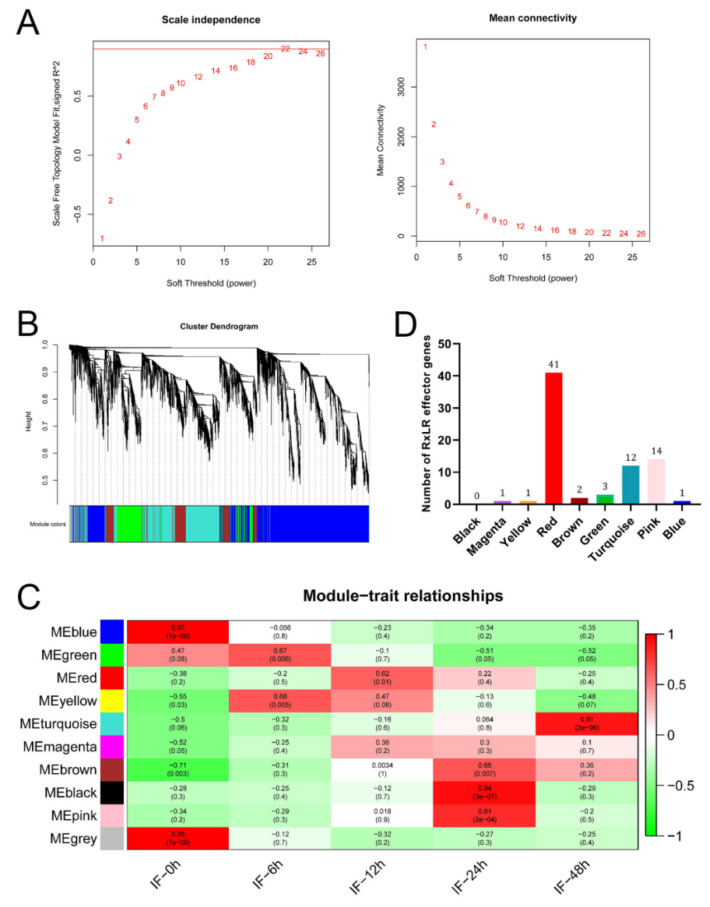
Construction of weighted gene co-expression network analyses (WGCNA) and module detection. (**A**) Analysis of the scale-free topology model fit index for soft threshold powers (β) and the mean connectivity for soft threshold powers. Left, the scale of the free fit index (*y* axis) as a function of the soft thresholding power (*x* axis). Right, the mean connectivity (degree, *y* axis) as a function of the soft thresholding power (*x* axis). The β value was set to ensure the high-scale independence (near 0.9) and low mean connectivity (near 0). (**B**) Module assignment in hierarchical clustered genes. Genes within different modules are labeled with different colors according to the WGCNA’s conventions. Ten co-expression modules were constructed and shown with distinct colors. The grey module contains all genes that were not involved in clustering and thus were not applied to subsequent analysis. (**C**) Matrix showing a module–trait relationship. Each row corresponds to a module. Each column corresponds to a time result. The module–trait relationship is colored based on their correlation, and red and green colors indicate strong positive and negative correlations, respectively. (**D**) Distribution of differentially expressed RxLR effectors in different modules. The number of RxLR effectors was counted after all modules filtered by the criteria that have weight value greater than 0.15.

To explore genes that regulated the expression of RxLR effectors during the early stage of infection, we performed a WGCNA by using the 8381 DEGs. WGCNA was designed to identify highly correlated gene clusters and relate them to biological traits [23]. It modularly investigates the co-expressed genes and extracts intramodular hub genes from system networks, increasing the sensitivity to recognize the potential of worthwhile targets for biological regulations [23]. A total of 10 modules were obtained using a soft threshold of 22 (Figure 2A,B). The expression level of RxLR effectors at 12 hpi was the highest during early infection, so we focused on the module which was closely related with 12 hpi. The module eigengenes showed that the red module had the highest correlation with 12 hpi (R^2^ = 0.62, *p* < 0.01) (Figure 2C). Interestingly, we found that 41 (44%) RxLR effector genes were clustered in the red module, which is much more abundant than those in other modules (Figure 2D). Collectively, these findings suggest that the red module is closely related to RxLR effectors.

**Figure 3:**
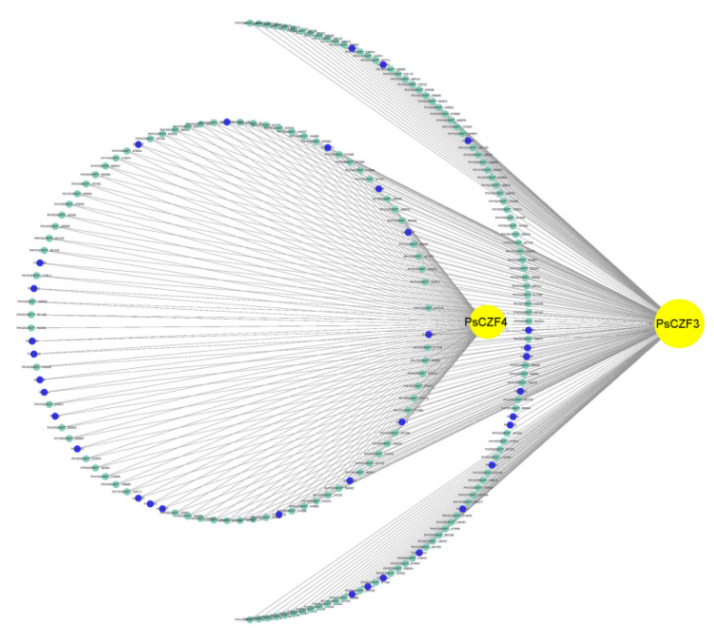
Network of transcription factors–effector interactions in the red modules. Yellow dots represent transcription factors, green dots indicate the correlated genes, and blue dots denote RxLR effector genes. The network was constructed by Cytoscape v3.7.2.

In the network of gene interactions, the key regulators, such as transcription factors, are usually at the center of the network regulation and are the ‘core’ of the study. Transcription factor genes with high connectivity represent essential genes that are the center of the network and might be involved in very important functions in co-expression networks. Therefore, the visualization of the co-expression network for the red module was constructed to explore high connectivity transcription factor genes by Cytoscape v3.7.2. Two putative transcription factor genes PHYSODRAFT_353615 and PHYSODRAFT_286006 were identified from the red module according to the Joint Genome Institute website (JGI) transcription factor database (https://mycocosm.jgi.doe.gov/mycocosm/proteins-browser/browse;p3YEuM?p=Physo3, accessed on 12 November 2013)). Pfam v32.0 (http://pfam.xfam.org/, accessed on 11 June 2020) analysis revealed that PHYSODRAFT_353615 contained C_2_H_2_-type zinc finger protein (ZFP) domains, while PHYSODRAFT_286006 possessed a C3HC4-type zinc finger (RING finger) domain. We named PHYSODRAFT_353615 as *PsCZF3* (Genbank accession number: XP_009516505.1), and PHYSODRAFT_286006 as *PsCZF4* (Genbank accession number: XP_009527011.1). In order to identify the genes regulated by the two transcription factors, we constructed a co-expression sub-network employing the two transcription factors as guide genes. We found that 197 genes were highly correlated with *PsCZF3*, and 94 genes were highly correlated with *PsCZF4*. To our surprise, the 94 genes belonged to the members of the 197 genes, and PsCZF4 was highly associated with PsCZF3 (Figure 3). Closer examination indicated that among the 197 genes, 35 RxLR effector genes were closely correlated with *PsCZF3*, including 20 that were correlated with *PsCZF4*. These findings suggest that *PsCZF3* and *PsCZF4* can be two important transcription factors related to the RxLR effector regulation during the early stage of infection.

**Figure 4:**
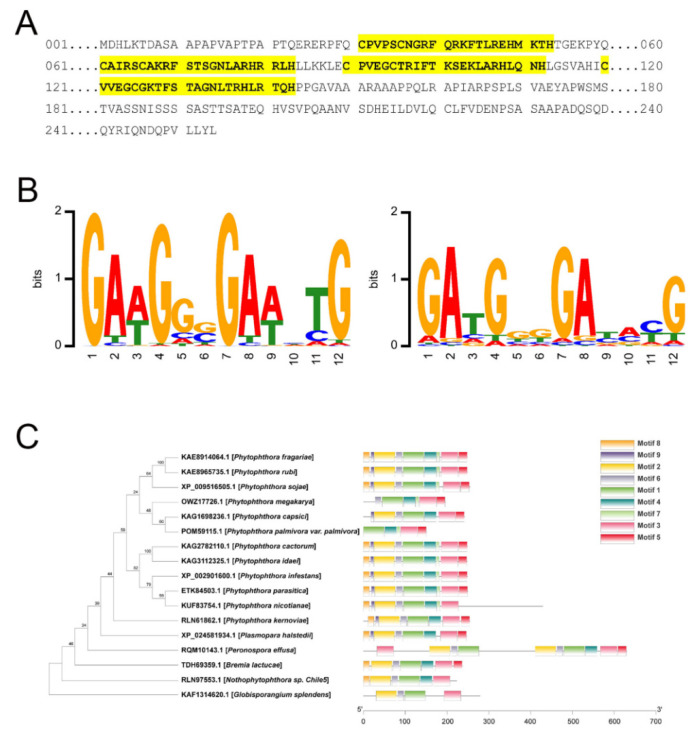
PsCzf3 protein domain analyses. (**A**) Amino acid sequence and putative C_2_H_2_-type zinc finger domains of PsCZF3. Amino acid sequences highlighted in yellow indicate predicted C_2_H_2_-type zinc finger domains. (**B**) Sequence logos for the predicted transcription factor binding site (TFBS) sequences. Left, TFBS analysis based on the webtool “ZFModels” (http://stormo.wustl.edu/ZFModels/, accessed on 1 April 2014); Right, TFBS prediction based on the webtool “DNA-binding Specificities of Cys2His2 Zinc Finger Proteins” (http://zf.princeton.edu, accessed on 9 September 2010). (**C**) Motif analysis of selected PsCZF3 orthologs across oomycetes. Each motif is represented by a numbered colored box. The length of the protein can be estimated while using the scale at the bottom.

As the connectivity of *PsCZF3* is higher compared to *PsCZF4* in the red module, we would like to focus on *PsCZF3*. Given that PsCZF3 is a C_2_H_2_-ZFP transcription factor, we further dissected its protein structure, particularly its DNA-binding sites. PsCZF3 is composed of 254 amino acids, and contains four conserved C_2_H_2_-type zinc finger domains (Figure 4A). The four zinc finger domains are the DNA binding domain of PsCZF3 and able to recognize 12-nucleotide DNA sequence (Figure 4B). We also examined the conservation of the PsCZF3 protein across other oomycete species, and found that the protein is highly conserved among 16 species in six genera of oomycetes (Figure 4C; Supplementary Table S4), indicating PsCZF3 may be a regulator that widely controls the expression of RxLR effector genes in different oomycete species.

**Figure 5:**
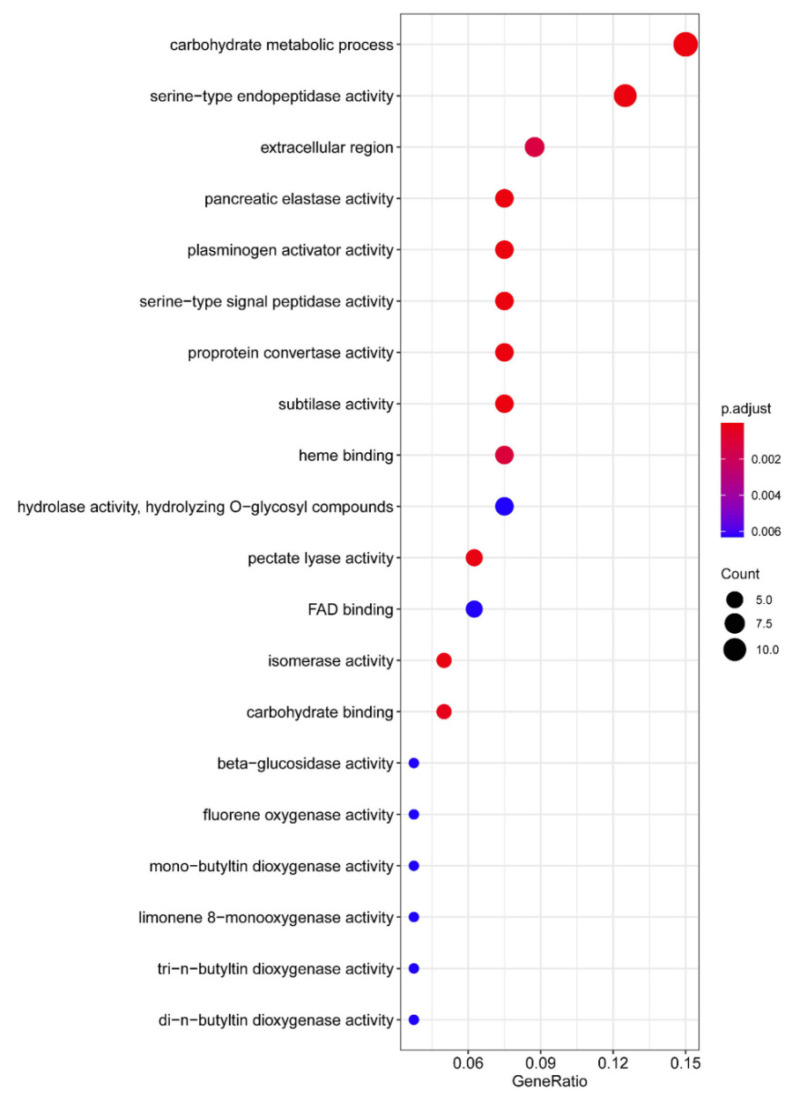
Gene Ontology (GO) enrichment analysis of the genes correlated with *PsCZF3*. Color scale represents the Benjamini–Hochberg adjusted *p*-values, and the size of circles indicates gene numbers. X-axis, GeneRatio, amount of DEGs enriched in the pathway/amount of all genes in annotation gene set; *Y*-axis, GO term (*p* < 0.05).

In addition to the RxLR effector family, we also noticed that other effector families are present in the aforementioned 197 gene pool, such as N-terminal YxSL[RK] (Tyr-Xaa-Ser-Leu [Arg/Lys]) motif containing secreted protein (YxSL) [30], protease, necrosis- and ethylene-inducing-like protein (NLP) [31], small cysteine-rich protein (SCP), elicitin, crinkler (CRN) and carbohydrate-active enzymes (CAZymes) (Supplementary Figure S3; Table S5). The gene ontology analysis of the genes that are highly correlated with *PsCZF3* revealed that CAZymes included the “carbohydrate metabolic process”, “hydrolase activity”, “hydrolyzing O−glycosyl compounds”, “pectate lyase activity” and “carbohydrate binding” (Figure 5). These enzymes are also secreted proteins that are mainly involved in the degradation of plant cell walls and promote adhesion, invasion, colonization and the nutrient absorption of pathogens [32,33]. These findings indicate that PsCZF3 may not only regulate RxLR effectors, but also regulate some carbohydrate-active enzymes during the early stage of infection.

## 4. Discussion

In this study, we demonstrated that WGCNA in combination with digital RNA-seq to illustrate the regulation network in oomycetes. WGCNA has been widely used for various genomic applications [34,35]. Unlike ordinary clustering methods, WGCNA uses correlation coefficient weighted values in its analysis, making the connections between genes in the network obey a scale-free network distribution, an algorithm that is more biologically meaningful. One of the biggest advantages of WGCNA is its ability to divide thousands of genes from multiple samples into several modules based on their expression patterns. By analyzing them on a module-by-module basis, the computational workload is decreased while the accuracy is increased. We discovered a C_2_H_2_-type zinc finger protein transcription factor that is associated with 35 RxLR effector genes and other virulence factors such as CAZymes. This study advances our understanding of transcriptional regulation in oomycetes.

In comparison to digital RNA-seq, the traditional RNA-seq method has some constraints, mainly due to the library construction step that relies on PCR amplification. Systemic errors could be magnified by increasing PCR cycles, which is more prominent in sequencing with the small initial samples. As a result, the sequencing outcomes sometimes do not truly reflect the expression levels of genes or transcripts in different samples. UID-mRNA-Seq is an absolute quantification of the initial molecule by counting UID, and therefore, more accurate gene expression results can be observed. In this study, 23,807 genes of *P. sojae* were detected in at least one library using digital RNA-seq. In the previous study, 14,969 genes of *P. sojae* were detected in at least one library using traditional RNA-seq [5]. The number of detected genes increased from 14,969 to 23,807, indicating that the transcriptome profiling by digital RNA-seq is not only more accurate but also more abundant. In the present study, a total of 316 RxLR effector genes were detected during the early infection stage of *P. sojae* to soybean leaves. The expression of these RxLR effectors was markedly up-regulated and reached the peak value at 12 hpi. The finding is consistent with the prior studies reported by Ye et al. [5], suggesting that 12 hpi is an important time point for RxLR effectors.

C_2_H_2_-type ZFP is the largest transcription factor superfamily in eukaryotes [36]. The only remarkable feature of the C_2_H_2_-type ZFPs is its DNA binding domain that contains zinc fingers arranged in tandem [37]. Each zinc finger is able to recognize 3-nucleotide DNA sequences [38], and with a combination of adjacent zinc fingers, C_2_H_2_-type ZFPs recognize long and complex DNA patterns. C_2_H_2_ transcription factors were shown to be important for pathogenic fungi, such as *Aspergillus fumigatus* [39,40], *Botrytis cinerea* [41], *Candida albicans* [42], *Cryptococcus neoformans* [43], *Ustilago maydis* [44] and *Magnaporthe oryzae* [45]. The disruption of C_2_H_2_ transcription factor in these fungi attenuated fungal virulence. In *P. sojae*, the C_2_H_2_ transcription factor *PsCZF1* was required for growth, development and pathogenesis. *PsCZF1* silenced transformants displayed a deficiency in terms of hyphal growth and sporulation, and loss of virulence on host soybean cultivars [46]. With the emerging CRISPR/Cas9 technology [47], it will be of interest to study the function of *PsCZF3* in the future.

## Data Availability

The RNA-Seq data are available in the NCBI BioProject database at https://www.ncbi.nlm.nih.gov/bioproject/with accession code PRJNA832871 (accessed on 27 April 2022).

## Supplementary Materials

The following supporting information can be downloaded at: https://www.mdpi.com/article/10.3390/jof8100998/s1, Figure S1: Confirmation of expression of 12 randomly selected DEGs by qRT-PCR; Figure S2: Average gene expression of the RxLR effector family during the early stage of infection; Figure S3: Gene categories correlated with *PsCZF3*; Table S1: Primers used in this study; Table S2: Statistical analysis of digital RNA-seq reads; Table S3: Summary of digital RNA-seq reads mapped to the reference genome; Table S4: *PsCZF3* ortholog genes in the oomycetes; Table S5: Protein-coding genes correlated with *PsCZF3.*
