# Supplementary material for: Identification of a C2H2 Transcription Factor (PsCZF3) Associated with RxLR Effectors and Carbohydrate-Active Enzymes in Phytophthora sojae Based on WGCNA
Source: J Fungi (Basel). 2022 Sep 22;8(10):998. doi: 10.3390/jof8100998 (PMC9605361; doi:10.3390/jof8100998)
Supplement: Supplementary file 1 [file jof-08-00998-s001.zip › Supplementary Table S1.pdf]

**Table S1 Primers used in the study**

| Name of gene | Name of primer | Nucleotide sequence (5′–3′) |
|---|---|---|
| PsAvr3a | Avr3a-F | CACGACTTCTGAGGGCCTAC |
| | Avr3a-R | AGCCTTCTTCTGGTATCTTTGGA |
| Avh1b-17 | Avh1b-17-F | GGCTGAACTCAACCACAAGC |
| | Avh1b-17-R | CGTCTTTTCGGCTCTCCAGT |
| Avh1b-15 | Avh1b-15-F | TCCCGACTTCGCCAAAAAGA |
| | Avh1b-15-R | CGGGTGGTACATGTCTGTGT |
| PsCZF4 | PsCZF4-F | GTGGCTCTACACGTTCGTCA |
| | PsCZF4-R | CGGTTCACCACGAACCTCTG |
| PHYSODRAFT_343410 | 343410-F | GTTCACCGTGGAGTCGCTTT |
| | 343410-R | GGTCGTCGCTGCCTGATGAA |
| PHYSODRAFT_488092 | 488092-F | GCCACAGAGAGTTGGACGAA |
| | 488092-R | TTGCCTTCAGTGTACGGGTC |
| PsCZF3 | PsCZF3-F | TTTGTTGACGAGAACCCCTCC |
| | PsCZF3-R | ACACAGGCTGGTCGTTCTG |

| | | |
|---|---|---|
| PHYSODRAFT_491221 | 491221-F | GGGCAAGTCGTCAAGTCCAT |
| | 491221-R | CCCAGAGTGAGCGTGTTTGA |
| PHYSODRAFT_306069 | 306069-F | TAATCGCGACTTCGGTTGCT |
| | 306069-R | TTCCTTGGTCGTCTTGGTCG |
| PHYSODRAFT_536186 | 536186-F | GCAACATGAGCGACACCATC |
| | 536186-R | GTGGGTGTGCAGGTATTCCA |
| PHYSODRAFT_520929 | 520929-F | GACGTGATTCTCCAGGACGG |
| | 520929-R | CCACGGATGGTTTTGTTGCC |
| PHYSODRAFT_337994 | 337994-F | GCGACTCCTACGATGCTCAA |
| | 337994-R | AGGTCGAGCTGGGTCATTTG |
| Actin | Actin-F | ACTGCACCTTCCAGACCATC |
| | Actin-R | CCACCACCTTGATCTTCATG |
