# Supplementary material for: Identification of a C2H2 Transcription Factor (PsCZF3) Associated with RxLR Effectors and Carbohydrate-Active Enzymes in Phytophthora sojae Based on WGCNA
Source: J Fungi (Basel). 2022 Sep 22;8(10):998. doi: 10.3390/jof8100998 (PMC9605361; doi:10.3390/jof8100998)
Supplement: Supplementary file 1 [file jof-08-00998-s001.zip › Supplementary Table S2.pdf]

Table S2 Statistical analysis of digital RNA-seq reads.

Sample	Raw_reads	Clean_reads	UID_reads	Clean_Q20(%)	Clean_Q30(%)	Clean_unique_Percentage	UID_unique_Percentage	Clean_GC(%)	UID_GC(%)	Clean2raw_read_ratio(%)
IF-0h_rep1	84663378	78563370	61988938	100	98.6	27.53	38.61	60.7	61.04	92.79
IF-0h_rep2	92239558	85847444	69031806	100	98.65	30.34	35.15	60.71	61.06	93.07
IF-0h_rep3	89623400	84672578	68090622	100	98.75	31.37	36.41	60.66	61	94.48
IF-6h_rep1	83528394	78276240	63690078	100	98.6	37.73	46.51	50.73	50.13	93.71
IF-6h_rep2	61716246	56720330	46541692	100	98.65	40.58	59.1	50.6	49.98	91.91
IF-6h_rep3	95722302	88227274	70134102	100	98.55	35.77	41.8	52.02	51.51	92.17
IF-12h_rep1	76304820	69322900	54815852	100	98.65	32.8	48.81	48.43	47.62	90.85
IF-12h_rep2	90992500	57929350	48100708	100	98.4	42.69	57.75	50.93	50.35	63.66
IF-12h_rep3	72869734	68488328	54943796	100	98.65	37.08	54.23	49.27	48.53	93.99
IF-24h_rep1	82863304	75245776	58392674	100	98.7	27.35	44.14	57.84	57.85	90.81
IF-24h_rep2	78848770	72626108	55652300	100	98.75	24.97	41.74	56.59	56.44	92.11
IF-24h_rep3	90179592	84472768	66530042	100	98.65	31.52	38.35	51.83	51.28	93.67
IF-48h_rep1	82078240	74010488	57096394	100	98.7	23.2	37.5	59.54	59.74	90.17
IF-48h_rep2	83701074	74633642	57854142	100	98.7	24.13	39.65	58.93	59.06	89.17
IF-48h_rep3	70456298	63627038	49182814	100	98.7	24.64	44.35	59.27	59.43	90.31
