# Supplementary material for: Identification of a C2H2 Transcription Factor (PsCZF3) Associated with RxLR Effectors and Carbohydrate-Active Enzymes in Phytophthora sojae Based on WGCNA
Source: J Fungi (Basel). 2022 Sep 22;8(10):998. doi: 10.3390/jof8100998 (PMC9605361; doi:10.3390/jof8100998)
Supplement: Supplementary file 1 [file jof-08-00998-s001.zip › Supplementary Table S3.pdf]

Table S3 Summary of digital RNA-seq reads mapped to the reference genome

Sample	total_reads	total_mapped(%)	non_unique(%)	unique(%)	un_mapped_reads(%)	read1(%)	read2(%)	reads_map_plus(%)	reads_map_minus(%)	non_splice_reads(%)	splice_reads(%)
IF-0h_rep1	61988938	60422158(97.47)	5801505(9.60)	54620653(90.40)	1566780(2.53)	27296965(49.98)	27323688(50.02)	27315583(50.01)	27305070(49.99)	49157498(90.00)	5463155(10.00)
IF-0h_rep2	69031806	67156708(97.28)	5797155(8.63)	61359553(91.37)	1875098(2.72)	30667194(49.98)	30692359(50.02)	30684315(50.01)	30675238(49.99)	55335066(90.18)	6024487(9.82)
IF-0h_rep3	68090622	66210313(97.24)	6761255(10.21)	59449058(89.79)	1880309(2.76)	29709020(49.97)	29740038(50.03)	29730172(50.01)	29718886(49.99)	53487305(89.97)	5961753(10.03)
IF-6h_rep1	63690078	61229365(96.14)	3265385(5.33)	57963980(94.67)	2460713(3.86)	28957897(49.96)	29006083(50.04)	28986060(50.01)	28977920(49.99)	43548698(75.13)	14415282(24.87)
IF-6h_rep2	46541692	45432767(97.62)	1375204(3.03)	44057563(96.97)	1108925(2.38)	22005312(49.95)	22052251(50.05)	22031245(50.01)	22026318(49.99)	33181639(75.31)	10875924(24.69)
IF-6h_rep3	70134102	67146772(95.74)	4110748(6.12)	63036024(93.88)	2987330(4.26)	31493600(49.96)	31542424(50.04)	31521861(50.01)	31514163(49.99)	48429035(76.83)	14606989(23.17)
IF-12h_rep1	54815852	52804439(96.33)	2583189(4.89)	50221250(95.11)	2011413(3.67)	25087720(49.95)	25133530(50.05)	25112052(50.00)	25109198(50.00)	36316750(72.31)	13904500(27.69)
IF-12h_rep2	48100708	47072433(97.86)	1410862(3.00)	45661571(97.00)	1028275(2.14)	22814745(49.96)	22846826(50.04)	22832401(50.00)	22829170(50.00)	34653968(75.89)	11007603(24.11)
IF-12h_rep3	54943796	53344808(97.09)	1756555(3.29)	51588253(96.71)	1598988(2.91)	25776166(49.97)	25812087(50.03)	25795931(50.00)	25792322(50.00)	38150570(73.95)	13437683(26.05)
IF-24h_rep1	58392674	56304461(96.42)	3508229(6.23)	52796232(93.77)	2088213(3.58)	26375912(49.96)	26420320(50.04)	26408888(50.02)	26387344(49.98)	45781646(86.71)	7014586(13.29)
IF-24h_rep2	55652300	53643244(96.39)	3096764(5.77)	50546480(94.23)	2009056(3.61)	25251272(49.96)	25295208(50.04)	25281158(50.02)	25265322(49.98)	42963573(85.00)	7582907(15.00)
IF-24h_rep3	66530042	64446331(96.87)	3377179(5.24)	61069152(94.76)	2083711(3.13)	30512380(49.96)	30556772(50.04)	30537893(50.01)	30531259(49.99)	47780432(78.24)	13288720(21.76)
IF-48h_rep1	57096394	54947177(96.24)	4064008(7.40)	50883169(92.60)	2149217(3.76)	25420409(49.96)	25462760(50.04)	25452052(50.02)	25431117(49.98)	45326741(89.08)	5556428(10.92)
IF-48h_rep2	57854142	55731886(96.33)	3912503(7.02)	51819383(92.98)	2122256(3.67)	25885916(49.95)	25933467(50.05)	25922489(50.02)	25896894(49.98)	45683024(88.16)	6136359(11.84)
IF-48h_rep3	49182814	47802248(97.19)	3062533(6.41)	44739715(93.59)	1380566(2.81)	22349934(49.96)	22389781(50.04)	22377617(50.02)	22362098(49.98)	39723713(88.79)	5016002(11.21)
