# Supplementary figures and images for: Identification of a C2H2 Transcription Factor (PsCZF3) Associated with RxLR Effectors and Carbohydrate-Active Enzymes in Phytophthora sojae Based on WGCNA
Source: J Fungi (Basel). 2022 Sep 22;8(10):998. doi: 10.3390/jof8100998 (PMC9605361; doi:10.3390/jof8100998)

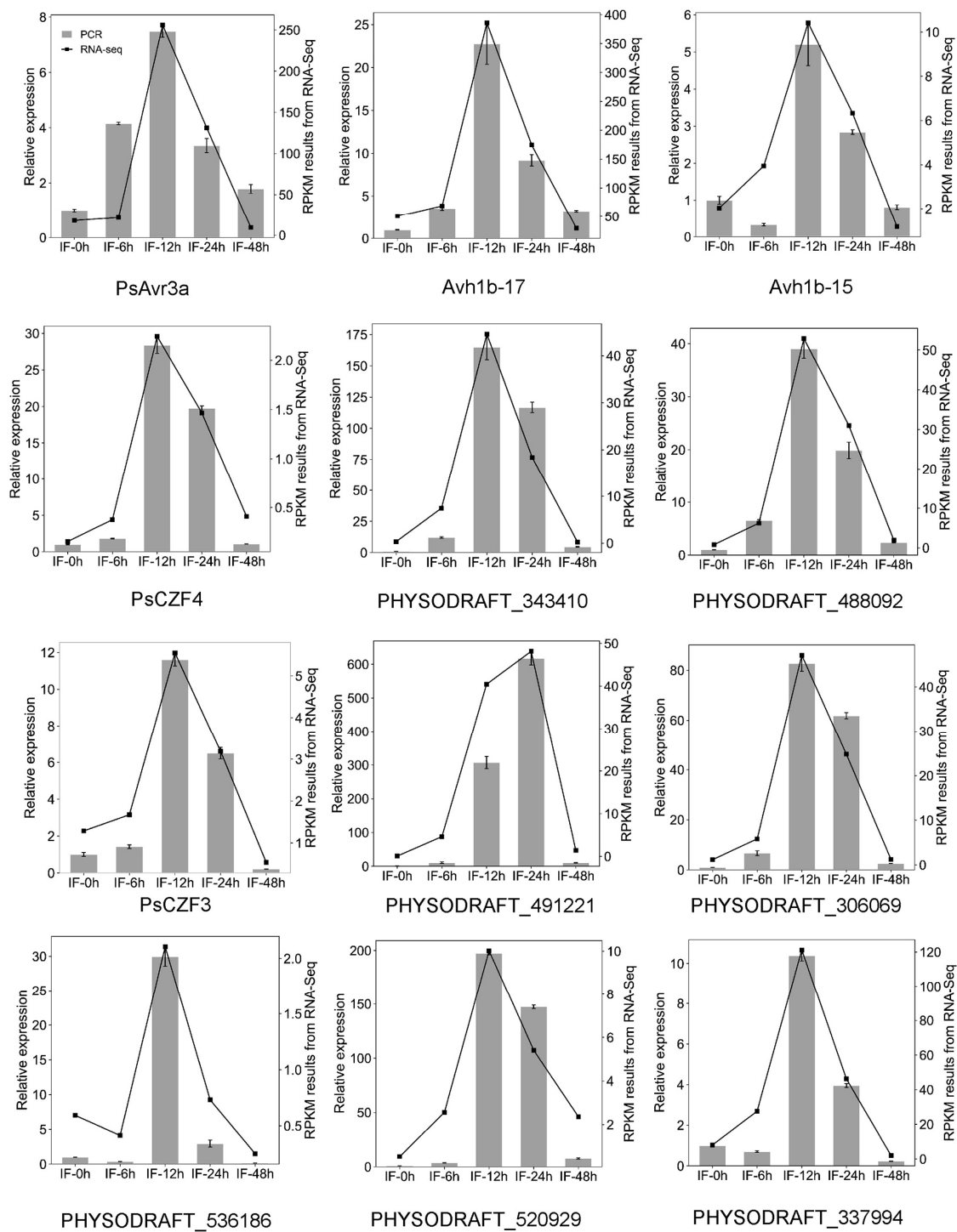

Figure S1

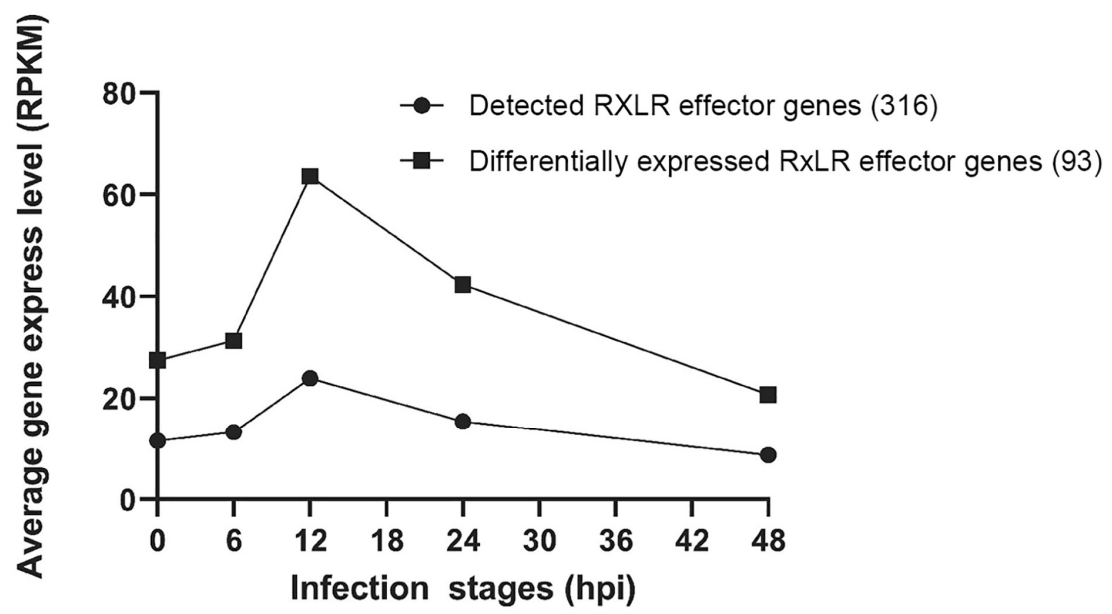

Figure S2

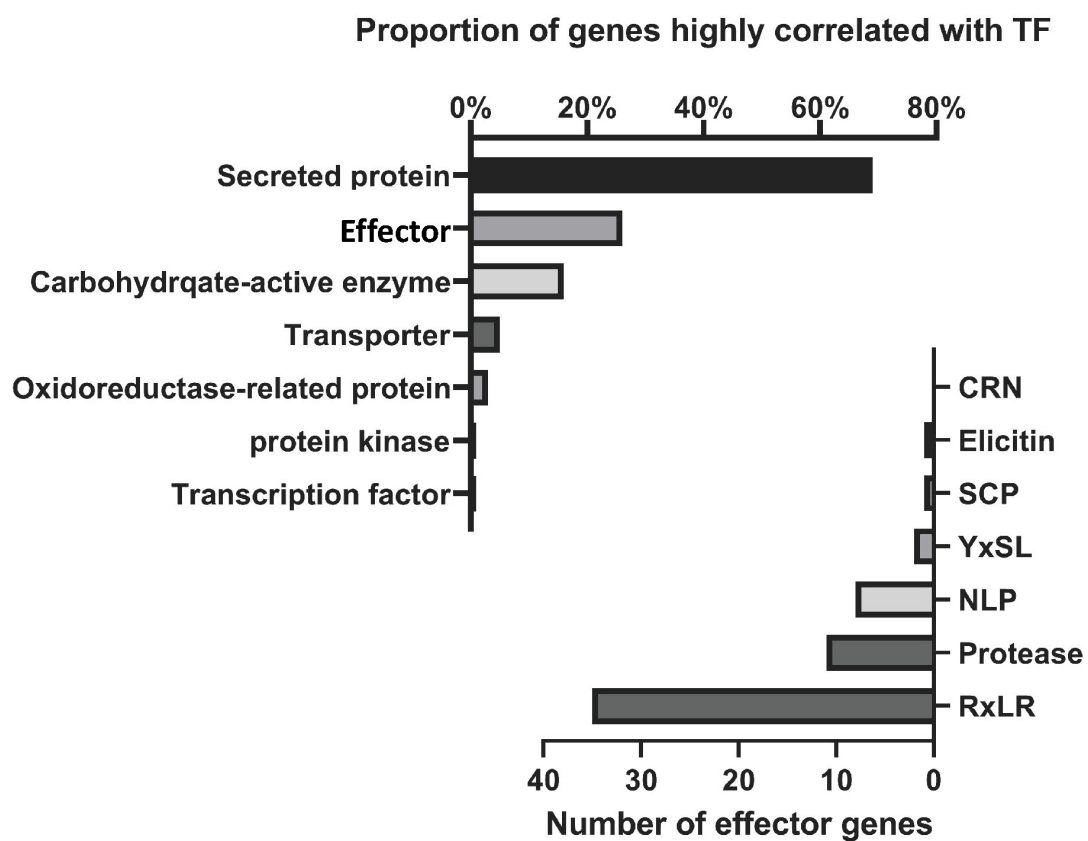

Figure S3

Supplement: Supplementary file 1 [file jof-08-00998-s001.zip › Supplementary Figure S1-S3.pdf]
